# Mining toxicogenomic data for dose-responsive pathways: implications in advancing next-generation risk assessment

**DOI:** 10.3389/ftox.2023.1272364

**Published:** 2023-11-17

**Authors:** A. Rasim Barutcu, Michael B. Black, Andy Nong

**Affiliations:** ScitoVation LLC, Durham, NC, United States

**Keywords:** NAM, transcriptomics, toxicogenomics (Tgx), BMD modeling, dose response

## Abstract

**Introduction:** While targeted investigation of key toxicity pathways has been instrumental for biomarker discovery, unbiased and holistic analysis of transcriptomic data provides a complementary systems-level perspective. However, in a systematic context, this approach has yet to receive comprehensive and methodical implementation.

**Methods:** Here, we took an integrated bioinformatic approach by re-analyzing publicly available MCF7 cell TempO-seq data for 44 ToxCast chemicals using an alternative pipeline to demonstrate the power of this approach. The original study has focused on analyzing the gene signature approach and comparing them to *in vitro* biological pathway altering concentrations determined from ToxCast HTS assays. Our workflow, in comparison, involves sequential differential expression, gene set enrichment, benchmark dose modeling, and identification of commonly perturbed pathways by network visualization.

**Results:** Using this approach, we identified dose-responsive molecular changes, biological pathways, and points of departure in an untargeted manner. Critically, benchmark dose modeling based on pathways recapitulated points of departure for apical endpoints, while also revealing additional perturbed mechanisms missed by single endpoint analyses.

**Discussion:** This systems-toxicology approach provides multifaceted insights into the complex effects of chemical exposures. Our work highlights the importance of unbiased data-driven techniques, alongside targeted methods, for comprehensively evaluating molecular initiating events, dose-response relationships, and toxicity pathways. Overall, integrating omics assays with robust bioinformatics holds promise for improving chemical risk assessment and advancing new approach methodologies (NAMs).

## Introduction

Transcriptomic data has proven to be highly informative in toxicology for determining cellular modes of action (MOA) and points of departure (POD) for hazard assessment and the comparative potency of congeners. MOA refers to a sequence of biological events and processes that occur in an organism upon exposure to a chemical or substance that leads to a particular toxic effect or adverse outcome. POD, on the other hand, is the reference point or a dose level on a dose-response curve at which a specific adverse effect or toxic response begins to occur. Community standards for best practices in analyzing transcriptomic data have observed significant convergence in principles for statistical analyses, use of benchmark dose (BMD) model fitting, and POD derivation (Farmahin et al., 2017; Johnson et al., 2022; National Toxicology Program, 2022). Analysis workflows for transcriptomic data have matured significantly in the past few years, and these activities can be standardized for most applications in toxicology. By automating much of the analytical processing, the efficiency and throughput of transcriptomic data analysis significantly increase, while the cost decreases. Automation in a computational pipeline also assures that community best practices are uniformly applied to data, ensuring that comparisons across compounds and experiments are unbiased in terms of quality control assessments, statistical analyses, and data normalization.

There is significant interest in using transcriptomics, particularly coupled with *in vitro* studies, to derive PODs as a means of reducing costs and time to completion for toxicology applications. With increasing interest in using transcriptomic data for the read-across of the MOA and potency of chemicals in cellular response to *in vivo* exposure, the use of standard transcriptomic analyses and relational databases for comparative analyses becomes essential (Raies and Bajic, 2016; Kleinstreuer et al., 2021). While much raw transcriptomic data is publicly available (in databases such as the NIH NCBI Gene Expression Omnibus), the analyzed results and their interpretation are not. This makes comparison with public or legacy data cumbersome, as often analyses need to be repeated from raw data for comparison with any new transcriptomic data. Addressing the computational uncertainty from transcriptomics tools is the first of many steps in the acceptance of NAMs for future risk assessments of compounds. Importantly, how different bioinformatic approaches affect the interpretation of toxicogenomics data, such as MOA or POD values, has not systematically been studied.

In this study, we compare different transcriptomic pipelines to capture both raw and final processed data used for the interpretation of MOA as well as POD values derived from BMD analysis and gene ontology (GO) pathway enrichment (Black et al., 2022). Our standardized process consists of quality control assessment of the raw dataset, RNA sequence differential gene expression analysis, BMD modeling of genes, and ontology enrichment of the gene related pathways for POD derivation. We performed comparative analysis of previously published (Harrill et al., 2021) and the current modeling approaches, and we identified several differences in BMD pathway- and gene-derived POD values of various compounds (Table 1).

**TABLE 1:** Flow Chart listing the similarities and differences of the bioinformatic approaches implemented in this study *versus* Harrill et al., 2021.

Harrill *et al*	This study
**Differential Gene Expression Analysis**	
• I FC I > 1.5, FDR <0.05 cut-off	• I FC I > 1.5, FDR <0.05 cut-off
• Single Sample Gene Signature Enrichment Analysis (ssGSEA) of chemical treatments	• I FC I > 2, FOR <0.01 cut-off
	• Analysis of overlap of common DEGs and associated pathways regulated by different chemicals
**BMD pathway Analysis**	
• BMD distribution of chemical signatures	• Systematic analysis of enriched BMD pathways across all chemicals
• Comparison of transcriptomic-derived biological pathway altering concentrations (BPACs)	• BMDL/BMD/BMDU and gene count distribution of significant pathways
• Focus on estrogen receptor (ER)-related pathways	• Assessment of pathway and gene-based BMD values of overlapping pathways
	• Focus on nitrogen metabolic processing pathway

While past studies have provided important insights by focusing on particular exposure-relevant processes like estrogen-receptor alpha (ERα) signaling pathway, our work aims to complement this understanding through a holistic systems biology approach. Therefore, in this study, we sought to ask how an unbiased and holistic analysis of transcriptomic data using integrated bioinformatic approaches could enhance our understanding of chemical toxicity, dose-response relationships, and toxicity pathways, and how this approach compares to traditional targeted methods in chemical risk assessment. By broadly profiling transcriptomic changes, we revealed additional pathways related to nitrogen metabolic processes that warrant further investigation. This unbiased, systemic perspective allows us to more fully appreciate the complex interconnectivity and breadth of biological impacts induced by chemical exposure. Furthermore, BMD modeling based on pathways rather than individual endpoints integrates signals across multiple affected systems to improve dose-response characterization. Overall, our unbiased system-wide analysis provides a multifaceted complement to enrich current knowledge of the diverse processes disrupted by these chemicals. By combining targeted mechanistic investigation with global omics profiling will lead to a comprehensive understanding of exposure effects across biological scales.

## Methods

### Extraction of publicly available RNA-seq dataset

The processed gene count data (Harrill et al., 2021) was downloaded from GEO Database with the accession number GSE162855 and can be found at the following link (https://www.ncbi.nlm.nih.gov/geo/query/acc.cgi?acc=GSE162855).

### Differential gene expression

Counted reads for each annotated transcript feature were analyzed for differential expression in R (v.3.5.3) using the BioConductor library DESeq2 (v.1.20.0) (Love et al., 2014). DESeq2 uses a dispersion correction of the count data based on the negative binomial distribution and a maximum likelihood model to impute the prior data distribution for statistical testing. DESeq2 employs a specialized Bayesian statistical approach to assess differential expression by simultaneously testing multiple pairwise comparisons of factors through the application of linear combinations. To avoid bias and unnecessary computation in the dispersion correction, the data is pre-filtered to exclude any annotated genomic feature for which there were no counts in any biological sample. DESeq2 is designed to otherwise deal with genes for which there are only counts in a very few or a minority of the samples when performing tasks such as FDR *p*-value corrections. The final output of DESeq2 is a table of estimated Log_2_ fold change, *p*-values for the defined contrasts tested, as well as Benjamini–Hochberg corrected false discovery *p*-values (FDR) In our analyses, we use as a statistical criterion, the Benjamini and Hochberg step-up false discovery rate corrected *p*-value of less than 0.05 (FDR<0.05) or less than 0.01 (FDR<0.01), and a change in expression (e.g., the ratio of the mean expression of treated samples relative to mean expression of control samples), up or downregulated genes in the compound-treated samples relative to controls of 1.5-fold (|FC|>1.5) or 2-fold (|FC|>2).

### Benchmark dose analysis

**FIGURE 1:**
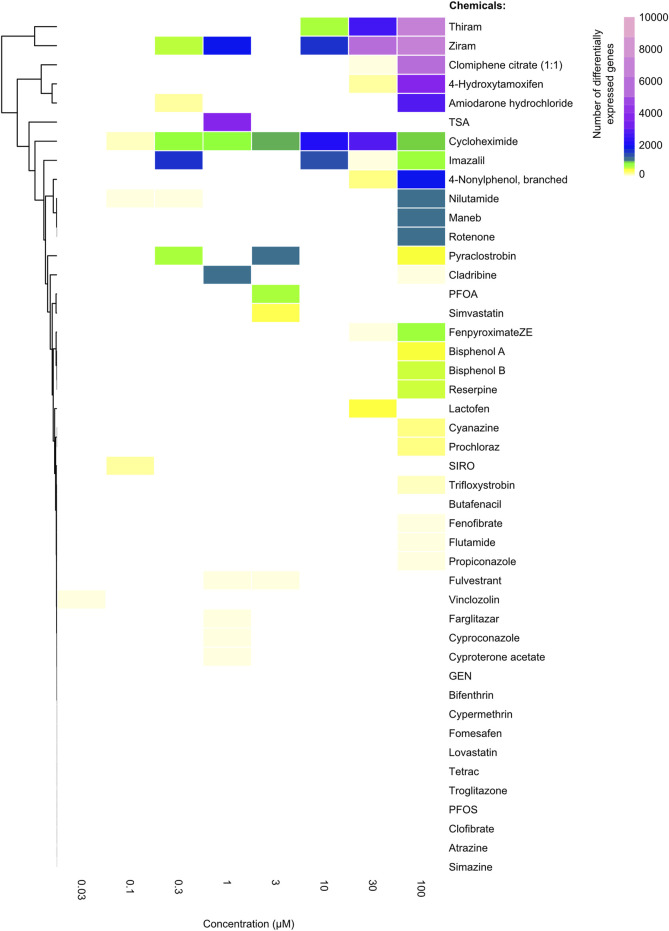
Heatmap showing the differentially expressed genes as a result of different chemical treatments. The number ofz differentially expressed genes for each chemical (rows) which were ordered based on unsupervised clustering at each drug concentration (columns). The color of each cell indicates the number of genes that were significantly differentially expressed (|FC| > 2, FDR <0.01) when compared to the DMSO (vehicle) controls. Ziram, thiram, clomiphene citrate (1:1), 4-hydroxytamoxifen and amiodarone hydrochloride displayed the largest number of differentially expressed genes at the highest dose, whereas chemicals such as atrazine and simazine did not display any differential gene expression.

Gene expression data were modeled using the BMDExpress software package (version 2.3, Build 3) (Phillips et al., 2019). Details of the BMDExpress framework are described in Black et al., 2022. First, normalized Log_2_-transformed expression values are computed for each gene in an RNA-Seq experiment. We have used the raw gene counts, which is the recommended data type to be used with DeSeq2 (Love et al., 2014), as an input for gene expression analysis. For stabilizing the transformation, we have added 0.1 to all the values in order to be able to handle zeroes in the dataset. Then, an independent gene pre-filtering, which is a part of the BMDExpress2 tool suite, is performed using a one-way ANOVA test. The ANOVA test is used to determine whether the responses to the various doses are all the same, which is the null hypothesis. With no restrictions on the direction of change of the responses, the alternative hypothesis for an ANOVA is that the responses are not all the same. The genes showing a |FC| > 1.5 and adjusted *p*-value <0.05 are retained for the BMD and the subsequent BMD pathway analysis. The filtered gene set is then fit to a series of dose-response models—typically the Hill model, Power model, linear, second-degree, and third-degree polynomials. Model selection follows a hierarchical approach. Hill models with a k-parameter estimate that is less than ⅓ of the lowest dose are excluded from best model consideration. Then, a nested likelihood ratio test is performed on the linear and higher-order polynomial models to select the best amongst the polynomial models run. The Akaike information criterion (AIC) for the selected best polynomial model is compared with the AIC for the power and non-excluded Hill models to select the best overall model, which is then used to calculate a BMD and benchmark dose lower confidence limit (BMDL). To avoid model extrapolation, genes with a BMD value greater than the highest concentration used in the experiment were removed from further analysis, as were poor-fitting best overall models with a goodness of fit *p*-value less than or equal to 0.1. The power parameter was restricted to >1 as values less than 1 have the potential to create models fit with a slope approaching infinity. We used 1 standard deviation (1 SD) BMR factor. A BMDU/BMDL ratio of greater than 40 is also grounds for the rejection of a best-fitting model, as this indicates an increasingly large 95% CI about the estimate. The final selection of genes with the best models passing these quality control thresholds can then be matched to elements in an ontology enrichment using the publicly available Gene Ontology Pathway database, including all the BP, CC and MF components (Ashburner et al., 2000). Various thresholds can be used to define the significant enrichment of a pathway. In this study, any ontology category with at least 5 or more best model elements found amongst the defined ontology category elements and with a Fisher’s exact test *p*-value <0.05 was considered enriched. We utilized the average BMDL, BMD, and BMDU values for assessment. BMDExpress2 is publicly available through a NIEHS repository (github.com/auerbachs/BMDExpress-2). The following criteria were used for filtering the BMD functional category results for all 47 chemicals tested: FDR<0.05; minimum number of genes observed in a pathway >5; GO category level = 2. The color scale of the heatmap in Figure 1 represents the number of dose-responsive genes observed in a pathway, and the sizes of the circles represent the -log2(FDR) values. The code for the BMD analysis can be found at https://github.com/rasimbarutcu/EPA_Harrill_etal_BMD_Reanalysis_Codes.git. GO analyses were performed by using the FuncAssociate 3.0 tool (Berriz et al., 2009). The bubble plots, which were generated using an available script (https://github.com/UBrau/GOplotTools) represent the FDR value and the number of genes identified for each pathway. All the figures in the manuscript were generated by using the R-ggplot2 package (Wickham, 2009).

## Results

### The extent of significantly differentially expressed genes

In order to interpret MOA as well as POD values derived from differential gene expression, BMD, and ontology pathway enrichment analyses, we re-analyzed the data from Harrill et al. (2021). The authors have screened 44 chemicals in MCF7 cells with 8 different concentrations to assess dose-dependent cellular response and generated highly reproducible RNA-seq data using the BioSpyder TempO-Seq hWTv1 assay (Yeakley et al., 2017). TempO-Seq is a gene expression profiling tool designed to monitor hundreds to thousands of genes at once in a high-throughput manner.

To assess the number of significantly differentially expressed genes per condition, as performed by Harrill et al., we conducted a pairwise DeSeq2 comparison for each dose of the drug-treated conditions, compared to untreated vehicle controls. Different from Harril et al., we performed two independent analyses utilizing two distinct thresholds for a gene to be considered significantly expressed. A more lenient threshold of Fold Change (FC) greater than 1.5, and a false discovery rate (FDR) (i.e., *p*-adjusted) value of less than 0.05, and a more stringent threshold of |FC| > 2 and FDR <0.01 were considered for a gene to be significantly differentially expressed (see Methods). A non-supervised hierarchical clustering of the chemicals based on the total number of DEGs for the two thresholds (i.e., both up- and downregulated genes) yielded highly comparable results with the published data, indicating the reproducibility of our pipelines (Figure 1). For instance, Thiram and Ziram, chemicals that inhibit metal-dependent and sulfhydryl enzyme systems, lead to the dysregulation of thousands of genes, whereas chemicals such as atrazine or simazine, which are used as herbicides inhibiting the photosystem II system, led to differential expression of a small number of genes (Figure 1; Supplementary Figure S1).

**FIGURE 2:**
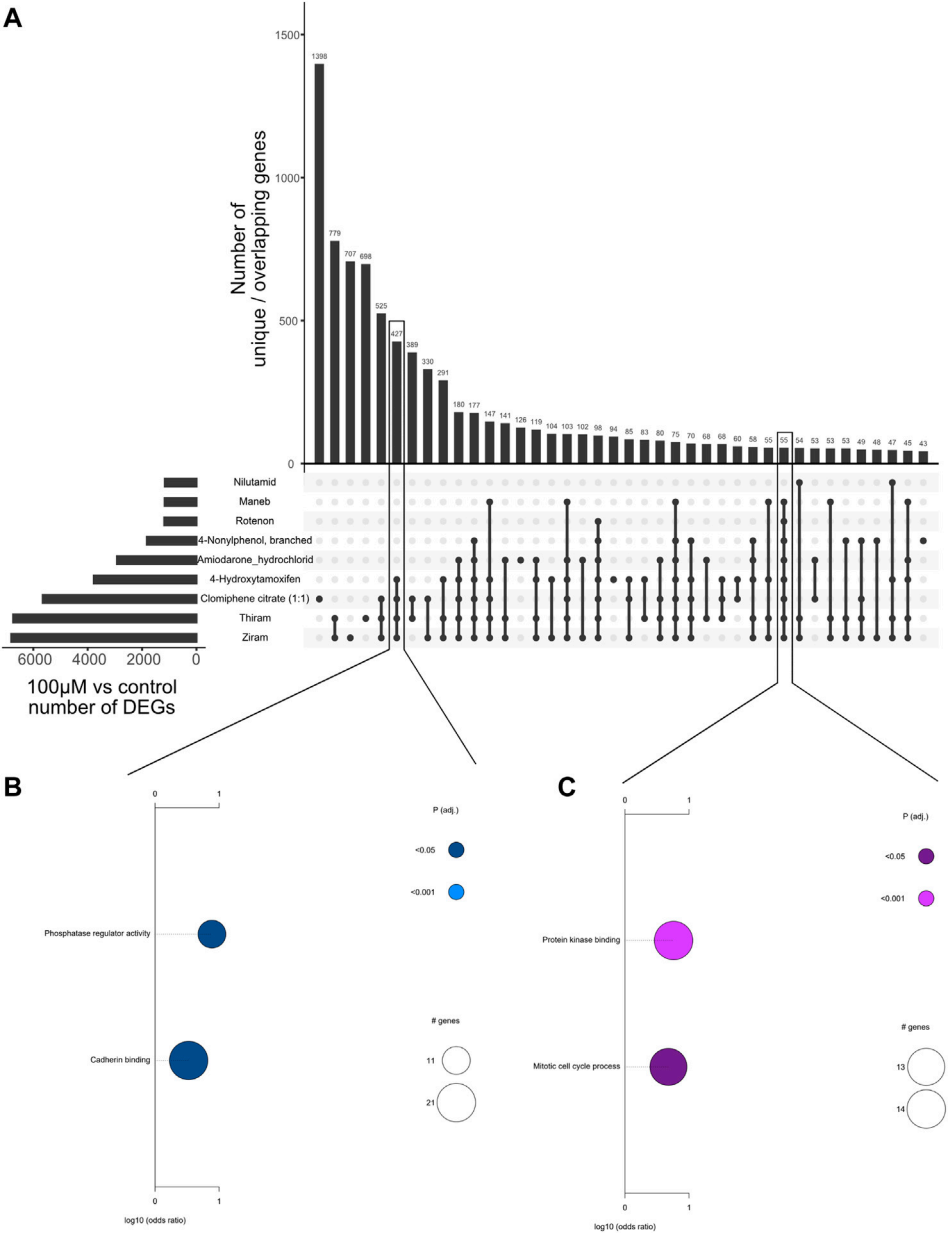
Overlap of differentially expressed genes upon treatment of different chemicals. **(A)** Upset plot showing the number of overlapping differentially expressed genes between different chemicals using the highest concentration *versus* DMSO control comparison. The overlaps are indicated by connecting dots below the bar graph, and the numbers on the bar graph indicate the number of overlapping genes. **(B)** Plot showing the significantly enriched Gene Ontology (GO) terms associated with the 427 genes commonly regulated between Ziram, Thiram, Clomiphene citrate (1:1), and 4-Hydroxytamoxifen. Pathways related with phosphatase regulator activity and cadherin binding are associated with these genes. **(C)** Plot showing the significantly enriched Gene Ontology (GO) terms associated with 55 genes commonly regulated between the 8 chemicals highlighted. Pathways related with protein kinase binding and mitotic cell cycle are associated with these genes.

Next, to assess the common genes that are differentially regulated upon treatment of each chemical, we generated an Upset plot which shows the number of differentially expressed genes (100 µM vs. control comparisons) that are unique to each chemical, or common between multiple chemicals (Figure 2A). Only the chemicals with the highest rate of overlaps are shown. We identified hundreds of genes that were commonly regulated between ziram, thiram, Clomiphene citrate (1:1), and 4-Hydroxytamoxifen (4HT), indicating that these drugs with different cellular effects can lead to the perturbation of similar transcriptional programs (Figure 2, see Discussion). Pathway analyses of these overlapping sets of genes suggest that several of these compounds regulate “phosphatase regulator activity,” or “cadherin binding”. Interestingly, 55 genes that were commonly regulated by 8 of the compounds were involved with ‘protein kinase binding’ and ‘mitotic cell cycle process’ (Figures 2B,C). These analyses therefore highlight the commonly regulated genes and pathways.

### Analysis of gene ontology pathways based on benchmark dose analyses

To assess the molecular, cellular, and biological pathways associated with drug-responsive genes, we implemented the BMDexpress workflow to derive the significant GO pathways from the transcriptomic data of each chemical (see Methods). Harril et al. have previously developed a gene expression signature-based concentration-response modeling approach, primarily focusing on pathways related to estrogen receptor bioactivity. As a complementary approach, we performed a series of systems-based analyses to assess pathway-based, as well as gene-based BMD and POD values in an unbiased way.

**FIGURE 3:**
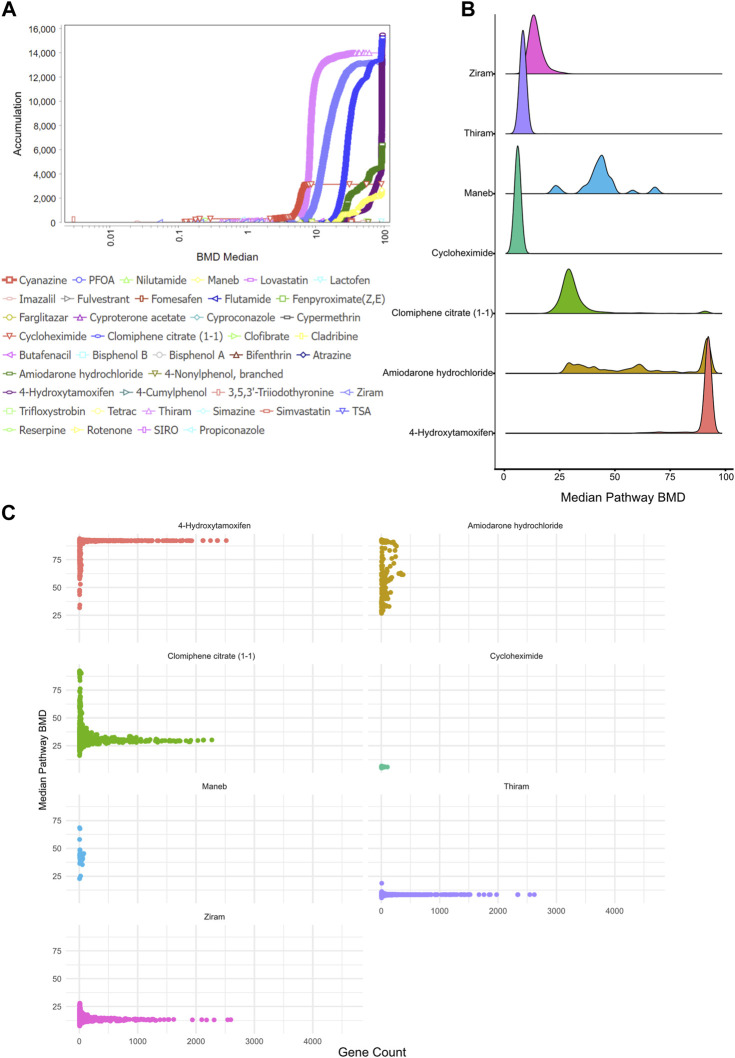
Benchmark dose (BMD) analysis **(A)** BMD Median Accumulation plot, which is a graphical representation of the distribution of BMD values for these pathways or endpoints, allowing one to assess the dose-response relationships and identify patterns or trends, for the genes in each tested condition. The ANOVA test (*p* < 0.05) and a fold-change of >1.5 or < -1.5 were used to pre filter the data. In BMDExpress v2.3, data were also post-filtered for best BMDU/BMD 40 and best fitPvalue >0.1. **(B)** Density plot showing the median pathway BMD levels for all significant BMD pathways for each chemical where a significant (FDR <0.05, number of genes >5) BMD pathway was observed. Ziram, thiram and cycloheximide showed a lower and narrower BMD distribution when compared to other chemicals, whereas maneb and amiodarone hydrochloride displayed a wider distribution of BMD values. **(C)** Scatter plot showing the number of genes as a function of median BMD value for the BMD pathways observed.

First, we visualized the BMD accumulation plots for pathways that have less than the maximum dose tested for each chemical (Figure 3A). A pathway was considered significant if it contained at least 5 genes and had a Fisher’s exact test *p*-value <0.05. This analysis demonstrated that 8 chemicals—4-Hydroxytamoxifen, Clomiphene citrate (1-1), Thiram, Ziram, Amiodarone hydrochloride, Cycloheximide, and Maneb—regulated significant numbers of BMD pathways (Figure 3A). We plotted the median BMD value of each pathway for each chemical to determine whether each chemical had a specific BMD value for the pathways it controlled. The results showed that certain chemicals, such as Maneb or Amiodarone hydrochloride, led to the regulation of certain pathways at various doses, while other chemicals, such as Ziram or Thiram, had specific BMD values for controlling the majority of BMD pathways (Figure 3B).

To gain further insight into the number of genes each pathway consisted of as a function of BMD values, we next plotted the number of genes per pathway across the BMD doses and identified that, consistent with the pathway BMD analysis (Figure 3B), a subset of pathways regulated by Ziram, Thiram, 4-Hydroxytamoxifen and Clomiphene citrate (1-1) showed a drastic number of genes, whereas pathways regulated by other compounds harbored a few number of genes (Figure 3C).

### Overlap of BMD pathways across the chemicals

**FIGURE 4:**
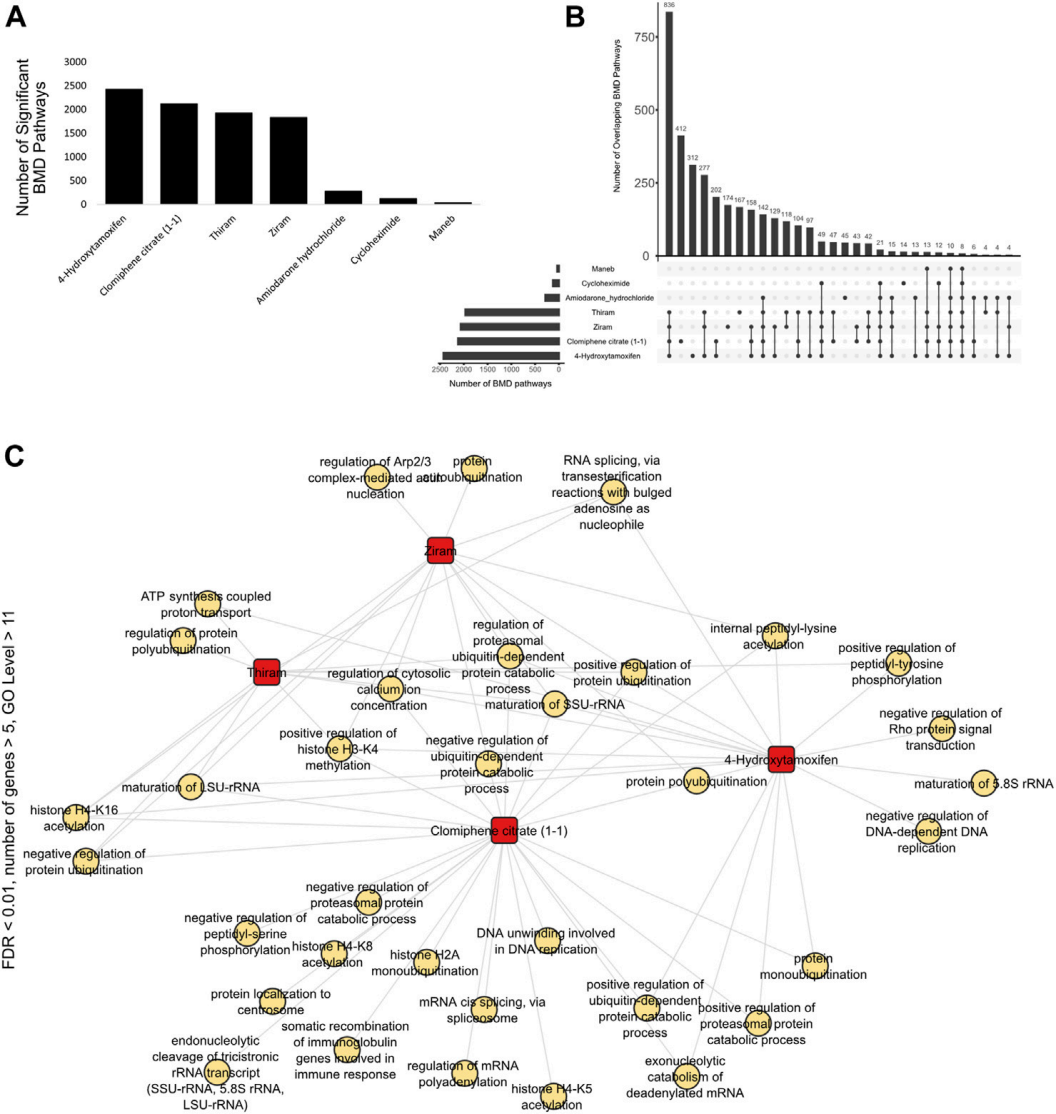
Overlap of BMD pathways **(A)** Bar graph showing the number of significant BMD pathways detected with each chemical using BMDExpress v2.3. **(B)** Upset plot showing the number of overlapping BMD pathways between different chemicals. The overlaps are indicated by connecting dots below the bar graph, and the numbers on the bar graph indicate the number of overlapping pathways. Similar to the overlap of differentially expressed genes (Figure 2A), ziram, thiram, clomiphene citrate (1:1), 4-hydroxytamoxifen and amiodarone hydrochloride displayed the largest number of BMD pathways. **(C)** Network graph showing the overlap of BMD pathways, pre-filtered with the threshold of FDR <0.01, number of genes >5, GO level >11, across the chemicals. There were several unique, as well as overlapping pathways. Overlapping pathways included terms such as “protein ubiquitination”, “histone H3-K4 methylation” and “regulation of cytosolic calcium ion concentration”.

We next analyzed the BMD pathways, defined as a pathway or biological process that is identified or characterized through Benchmark Dose (BMD) modeling analysis, that were commonly shared among all the cell type vs. chemical conditions and identified that, consistent with the DEG overlap analyses (Figure 2A), Ziram, Thiram, 4-Hydroxytamoxifen and Clomiphene citrate (1-1) displayed the highest number of overlapping BMD pathways (Figures 4A,B).

By visualizing the pathways based on the thresholds mentioned above, and by further filtering the data to include only GO category level 11 and above pathways and have a more stringent value FDR value <0.01, we identified that genes that are dose-responsive to thiram, ziram, Clomiphene citrate (1-1) and 4-Hydroxytamoxifen, which is a selective estrogen receptor modulator (SERM) and acts as an agonist or antagonist depending on the target tissues (Lonard et al., 2004), shared pathways such as “positive regulation of histone H3-K4 methylation,” “regulation of calcium ion concentration,” or “positive regulation of protein ubiquitination” (Figure 4C).

**FIGURE 5:**
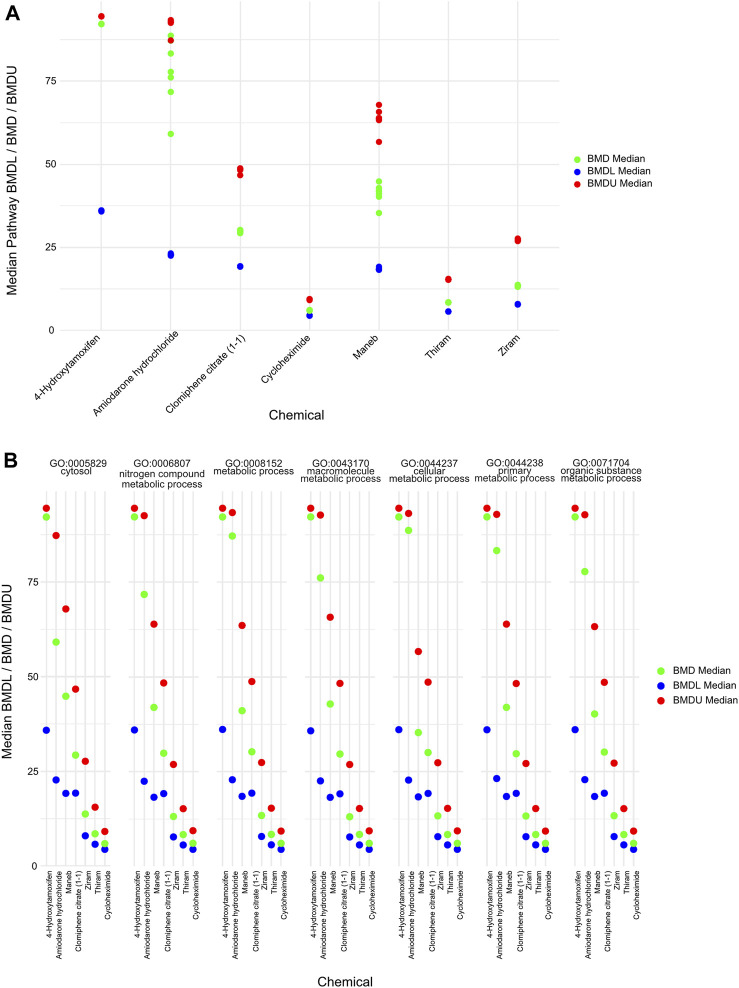
BMDL/BMD/BMDU values of overlapping pathways derived **(A)** Plot showing the average BMDL, BMD and BMDU values derived from overlapping BMD pathways for the chemicals in which there was a significant GO term. The BMD levels of certain chemicals such as amiodarone hydrochloride displayed a larger distribution than others such as ziram. **(B)** Plot showing the pathway-specific BMDL, BMD and BMDU values derived from each of the overlapping BMD pathways.

Moreover, to gather information about the BMD values that are related to different biological pathways, we plotted the lower BMD (BMDL), BMD and upper BMD (BMDU) values derived from the average of the significant GO pathways that were shared among the chemicals (Figure 5A). Furthermore, we plotted the individual pathway BMD values for the 7 overlapping pathways across the chemicals, and we identified similar ordering of the chemicals by mean BMD (Figure 5B). Importantly, we identified BMD values which—although similar in terms of ranking the chemicals by mean BMD values - are orders of magnitude higher than those observed by Harrill et al. (2021) where the authors derived the BMD values based on gene-level quantifications and gene signatures of biological pathway altering concentrations (BPACs). The source of these discrepancies is further discussed below (see Discussion). As a result, our findings indicate that BMD analysis derived from GO enrichments can yield different results based on which level the BMD values are extracted from (I.e., genes *versus* pathway levels). The ranking (from low to high values) of only a subset of the chemicals remains similar based on both our and Harrill et al. BMD approaches. Since our and Harrill et al. classification from DGE and BMD steps have some similarities, it is the final derivation of POD based on ontogeny which likely contributed to the different final quantified outcomes.

### Analysis of gene-level BMD values

Next, among the overlapping pathways, we have decided to focus on the “nitrogen metabolic processing” pathway (GO:0006807), since this process plays an extensive role in several carcinogenic and immune-related processes (Kurmi and Haigis, 2020; Zhang et al., 2022). In addition, nitrogen metabolism is essential for the biosynthesis of nucleotides, amino acids, and neurotransmitters (Brosnan, 2003; Wu, 2021). Disruption of this process can affect cell growth, protein synthesis, and nervous system function. Furthermore, chemicals that cause nitro-oxidative stress can impair nitrogen metabolism enzymes through protein damage or cofactor depletion. This leads to the buildup of ammonia and reactive nitrogen species, further amplifying cellular damage (Pacher et al., 2007). Nitrogen oxides generated during inflammation and immune responses to toxins can impact nitrogen metabolic processing. This interplay is important in chemical-induced immunotoxicity (Miller and Grisham, 1995). Finally, changes in nitrogen utilization and excretion in response to chemical exposures can serve as key event biomarkers indicating disruption of normal nitrogen homeostasis and variations in nitrogen metabolic genes may contribute to individual susceptibilities to certain chemical toxicities. Taken together, given the fundamental role of nitrogen metabolism in mediating biosynthesis, oxidative stress, immunotoxicity, and overall cellular homeostasis, characterization of the nitrogen metabolic processing pathway offers critical insights into mechanisms of toxicity and dose-response relationships for adverse chemical exposures.

**FIGURE 6:**
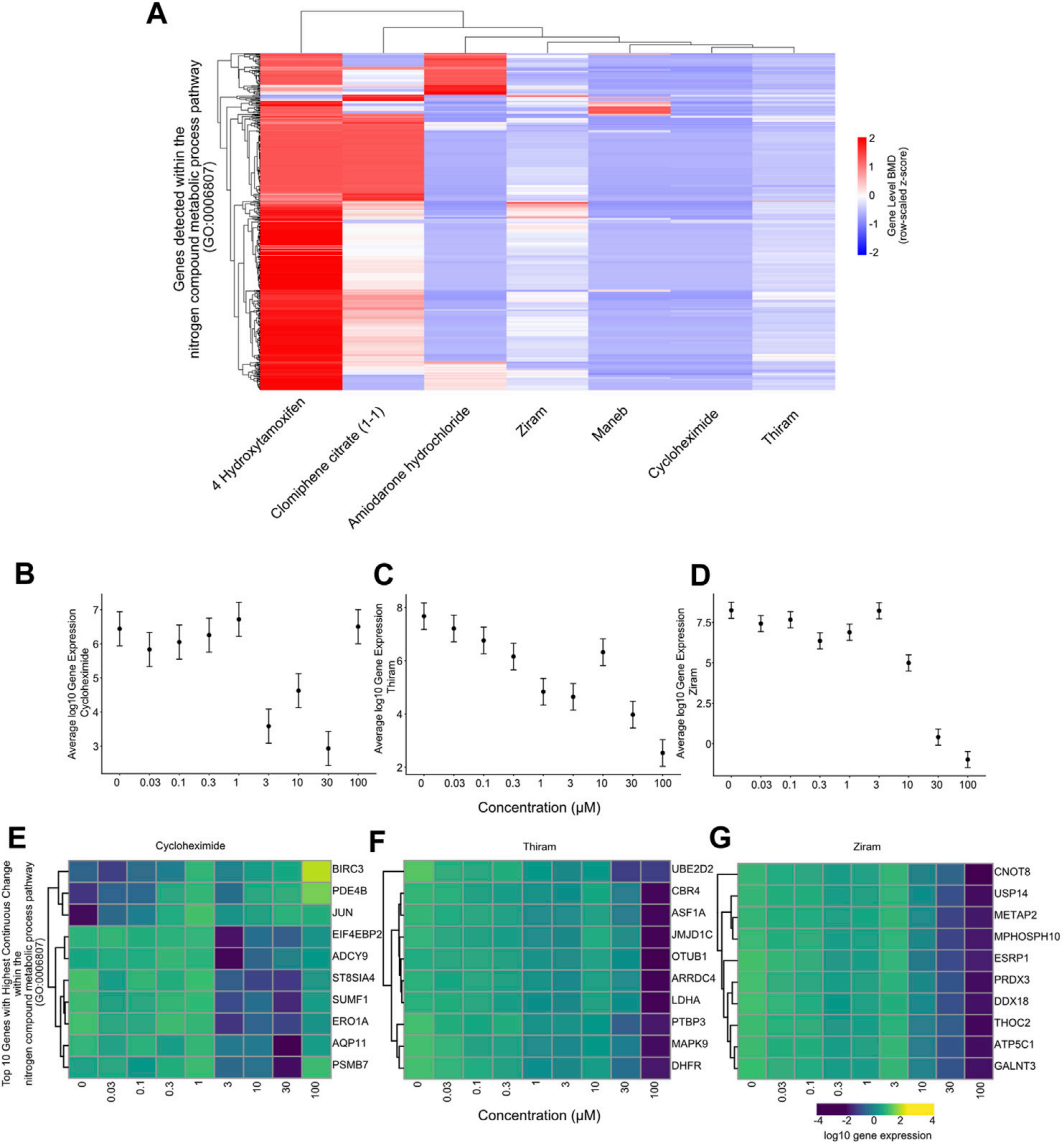
Gene-level BMD analysis for the nitrogen compound metabolic process pathway **(A)** Heatmap showing unsupervised hierarchical clustering of gene-level BMD patterns between genes within the nitrogen compound metabolic process pathway for each chemical. Scale: BMD values expressed as a z-score. The data suggests that different subsets of genes within the same pathway harbor distinct BMD levels among the chemicals. **(B)** Plot showing the log10 average gene expression of the genes within the nitrogen compound metabolic process pathway across the doses tested for Cycloheximide treated samples. **(C)** Plot showing the log10 average gene expression of the genes within the nitrogen compound metabolic process pathway across the doses tested for Thiram treated samples. **(D)** Plot showing the log10 average gene expression of the genes within the nitrogen compound metabolic process pathway across the doses tested for Ziram treated samples. **(E)** Heatmap showing the log10 gene expression values of the top 10 genes within the nitrogen compound metabolic process pathway which show the highest rate of regulation across the doses tested for Cycloheximide treated samples. **(F)** Heatmap showing the log10 gene expression values of the top 10 genes within the nitrogen compound metabolic process pathway which show the highest rate of regulation across the doses tested for Thiram treated samples. **(G)** Heatmap showing the log10 gene expression values of the top 10 genes within the nitrogen compound metabolic process pathway which show the highest rate of regulation across the doses tested for Ziram treated samples.

We first plotted the gene-level median BMD values for each gene in the nitrogen metabolic pathway for each chemical and have identified that different sets of genes within this pathway harbor varying levels of median BMD values. The result indicates that although different chemicals lead to downregulation of the “metabolic nitrogen processing” pathway, it does so by regulating different sets of genes from the same pathway (Figure 6A).

**FIGURE 7:**
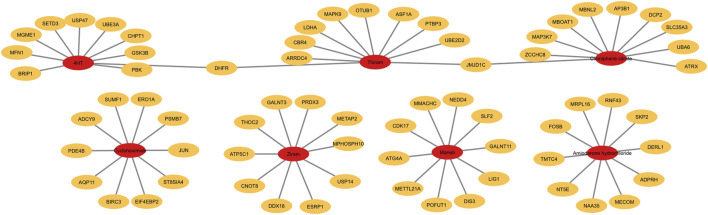
Top 10 genes within the nitrogen compound metabolic process pathway regulated by each chemical. Graph showing the top 10 genes within the nitrogen compound metabolic process pathway that are most drastically regulated across the doses.

We next plotted the average gene expression profile of the genes within this pathway as a function of dose for the chemicals with the lowest 3 median BMD values, Thiram, Ziram and Cycloheximide, and identified a consistent downregulation of these genes (Figures 6B–D). By selecting the top 10 most-regulated genes in terms of differential gene expression between the lowest and highest chemical doses, we plotted the gene expression levels and, consistent with all the genes in the pathway, we identified a consistent downregulation, and in some cases, upregulation of genes such as BIRCH3, PDE4B or JUN with Cycloheximide (Figures 6E–G). Finally, plotting the top 10 gene lists within the “metabolic nitrogen processing” pathway together for each chemical, we identified that each chemical regulates a distinct set of genes within this pathway (Figure 7). This chemical-specific gene expression signature within a shared perturbed pathway highlights the potential for toxicogenomic profiling to elucidate both common and unique mechanisms of action across related toxicants.

We also adopted two additional strategies to interpret the functional categories with regards to BMD values. In the first, the mean BMD of each pathway is plotted along the lowest 20, 10, or 5 BMD GO pathways. The second method, which we have previously implemented in Black et al., 2022, involves plotting the mean BMD values of each gene within the top 20, 10, or 5 lowest BMD pathways (Figure 5; Supplementary Figure S3). Our findings, particularly the gene-based analyses, point to the possibility that some chemicals, like Maneb, contain two populations of genes with distinct BMD values, as suggested by the bimodal distribution. This result is in line with the quantity of active signatures discovered by Harril et al., who also found that drugs like Fulvestrant and Cycloheximide have a bimodal distribution with some signatures having lower BMD values.

## Discussion

Toxicogenomics is emerging as a valuable systems science approach to inform chemical risk assessment across diverse applications. By providing insights into molecular mechanisms of biological effects, toxicogenomic profiling can enhance hazard characterization and derive points of departure to support safety determinations. Furthermore, the increasing interest in new approach methodologies (NAMs) has motivated efforts to establish robust and reproducible computational pipelines to analyze these complex genomics datasets. However, the field currently lacks consensus regarding optimal bioinformatics strategies, especially for benchmark dose modeling and derivation of points of departure from transcriptomics data. To address this need, we leveraged a recently published toxicogenomics dataset assessing dose-responses for 44 chemicals in MCF7 cells (Harrill et al., 2021). By performing complementary systems-level analyses using our validated pipeline (Black et al., 2022), we aimed to evaluate concordance and provide a reproducible workflow for pathway-based benchmark dose modeling from toxicogenomic dose-curves. Our unbiased characterization of differentially expressed genes, enriched ontologies, and pathway dose-dependencies highlights the power of holistic transcriptomic profiling to elucidate mechanisms of action and biological point of departures. Overall, this case study supports the reliability of our bioinformatics approach, while demonstrating the utility of toxicogenomics and robust computational methods to enhance chemical safety assessment.

Our re-analysis of the TempO-seq dataset from Harrill et al. provides important insights into the diversity of transcriptional responses induced by different chemical exposures. While some compounds like the herbicides atrazine and simazine elicited minimal gene expression changes, others including the dithiocarbamate fungicides thiram and ziram (Fanjul-Bolado et al., 2020) dysregulated thousands of genes. PFOA and simvastatin showed a relatively small number of differentially expressed genes at 3 µM, however they did not show any gene expression at higher doses. This may be either due to the saturation effect, where at higher doses, the biological systems or molecular pathways affected by the chemical may become saturated. This means that the maximum response or effect has already been achieved at lower doses, and further increases in dose do not lead to additional changes in gene expression. Interestingly, we identified hundreds of genes commonly perturbed across distinct chemicals such as the endocrine disruptors clomiphene and 4-hydroxytamoxifen. Pathway analysis revealed these shared gene expression signatures were enriched for processes like cell cycle regulation. This suggests certain core pathways may respond in a stereotypical manner to cellular stress, irrespective of whether the initial molecular initiating event is mitochondrial dysfunction, hormone receptor antagonism, or electrophilic reactivity. However, our Upset plot also indicated predominantly unique transcriptional profiles for most chemicals. Overall, these findings reveal both conserved and divergent transcriptomic effects across exposures, highlighting the value of toxicogenomic profiling for elucidating mechanisms of action. Our benchmark dose modeling based on pathways rather than individual genes leverages these shared and unique expression patterns to improve dose-response assessment.

We have identified that tens to hundreds of differentially expressed genes that are associated with “mitotic cell cycle process,” “phosphatase regulator activity” and “protein kinase binding” overlap across the chemicals at the highest 100 µM dose (Figure 2A). It is important to note that at concentrations as high as 100 µM, many chemicals are expected to elicit cytotoxic effects, which can overshadow the underlying biological mechanisms operative at lower, more biologically relevant concentrations. This may explain the observed overlap in gene expression profiles related to the “mitotic cell cycle process” ontology, as cytotoxicity-induced cell cycle arrest is a common outcome. Similar to the “mitotic cell cycle processes,” “protein kinase binding” and “phosphatase regulator activity” pathways play a crucial role and are biomarkers for cellular stress (Hotamisligil and Davis, 2016). Indeed, when we examine the individual genes overlapping between the 8 chemicals in Figure 2A, several genes, such as BCL2L11, AKT1, DDIT3, GADD45A, VEGFA, MAPK1, and JUNB, all of which are related to the kinase pathways, are found, indicating that these pathways may relate to chemical-induced cytotoxicity.

Our unbiased BMD modeling of pathways reveals both shared and unique dose-responsive profiles across diverse chemical exposures. The striking perturbation of numerous pathways enriched for processes like cell cycle regulation highlights the power of omics data to capture system-wide effects missed by individual endpoint analysis. Furthermore, the chemical-specific pathway modulation underscores the potential to leverage transcriptomics to delineate mechanisms of action and address the long-standing challenge of heterogeneity in dose-response. By objectively integrating signals across the inter-connectome, toxicogenomic BMD analyses offer a robust data-driven approach to chemical safety evaluation.

By performing an in-depth analysis of the nitrogen metabolic processing pathway, our study demonstrates the value of toxicogenomics for elucidating chemical mechanisms of action. While this fundamental pathway was perturbed across several compounds, our gene-level modeling and expression plots revealed chemical-specific BMD values and transcriptomic signatures. These nuances underscore the complexity hidden within shared perturbed pathways, and the need for systems-level perspectives.

It is important to note that in toxicity risk assessment, bioinformatics approaches play a crucial role (Stucki et al., 2022), however, one should keep in mind that they should be viewed as prioritization methods for further toxicity testing rather than standalone tools for definitive risk assessment (Harrill et al., 2019). Although bioinformatics approaches provide valuable insights into the potential toxicity of chemicals, elucidate underlying molecular mechanisms, and help identify potential hazards (Joseph, 2017), they have certain limitations that necessitate follow-up *in vitro* or *in vivo* research to establish concrete risk assessments (Andersen et al., 2019). For instance, if a chemical exposure leads to the dysregulation of genes, or BMD pathways or PODs associated with oxidative stress response, it suggests that the compound may induce oxidative damage. However, this hypothesis warrants further experimental testing. In addition, when dealing with a large number of chemicals, bioinformatics methods can help prioritize which ones warrant further investigation. If a transcriptomic analysis suggests that a chemical may disrupt key cellular pathways linked to adverse health effects, it becomes a higher priority for more detailed toxicological evaluation. In addition, using the average BMD values across overlapping BMD pathways (Figure 5) has certain limitations, as it may dilute the signals from the most sensitive pathways and miss unique molecular initiating events. Finally, transcriptomic data alone cannot capture all aspects of toxicity, such as non-genomic responses, epigenetic changes, or post-translational modifications (Baccarelli and Bollati, 2009; Lee, 2013; Tretyakova and Wang, 2018). Therefore, further experimental assays may be needed to validate findings and assess additional facets of toxicity.

## Conclusion

Overall, coupling unbiased pathway analysis with targeted follow-up of key processes provides multilayered insights into the molecular initiating events and dose-dependencies of adverse effects. Our integrated approach therefore leverages the depth of omics data to link pathway perturbations to underlying genomic biomarkers and biological mechanisms. Looking forward, systematic application of these bioinformatics workflows will add enhanced resolution of pathway dose-response, improving chemical risk assessment and advancing mechanistic toxicology.

## Data Availability

Publicly available datasets were analyzed in this study. This data can be found here: The processed gene count data (Harrill et al. 2021; PMID:33538836) was downloaded from GEO Database with the accession number GSE162855 and can be found at the following link (https://www.ncbi.nlm.nih.gov/geo/query/acc.cgi?acc=GSE162855).

## Supplementary material

Supplementary Figure S1 Heatmap showing the differentially expressed genes as a result of different chemical treatments. The number of differentially expressed genes for each chemical (rows) which were ordered based on unsupervised clustering at each drug concentration (columns). The color of each cell indicates the number of genes that were significantly differentially expressed (|FC| > 2, FDR < 0.01) when compared to the DMSO (vehicle) controls.

Supplementary Figure S2 Pathway-and gene-based BMD values. Boxplot showing the best BMD value distributions for the **(A)** lowest 5 pathways, **(B)** lowest 10 pathways. Ridge plots showing the best BMD value distributions per gene for the **(C)** lowest 5 pathways and **(D)** lowest 10 pathways.
