# Supplementary figures and images for: Mining toxicogenomic data for dose-responsive pathways: implications in advancing next-generation risk assessment
Source: Front Toxicol. 2023 Nov 17;5:1272364. doi: 10.3389/ftox.2023.1272364 (PMC10691261; doi:10.3389/ftox.2023.1272364)

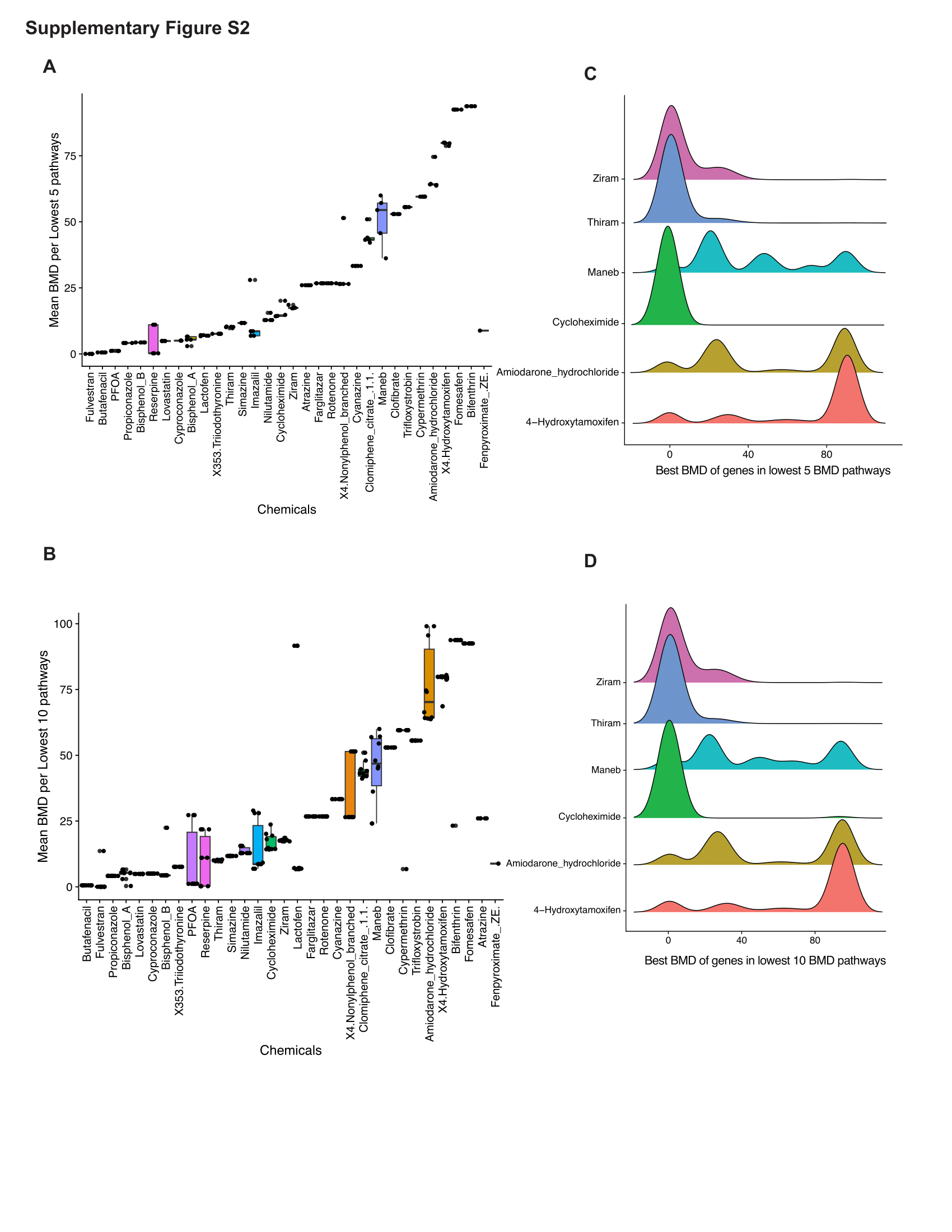

Supplement: Supplementary file 1 [file Image2.PNG]

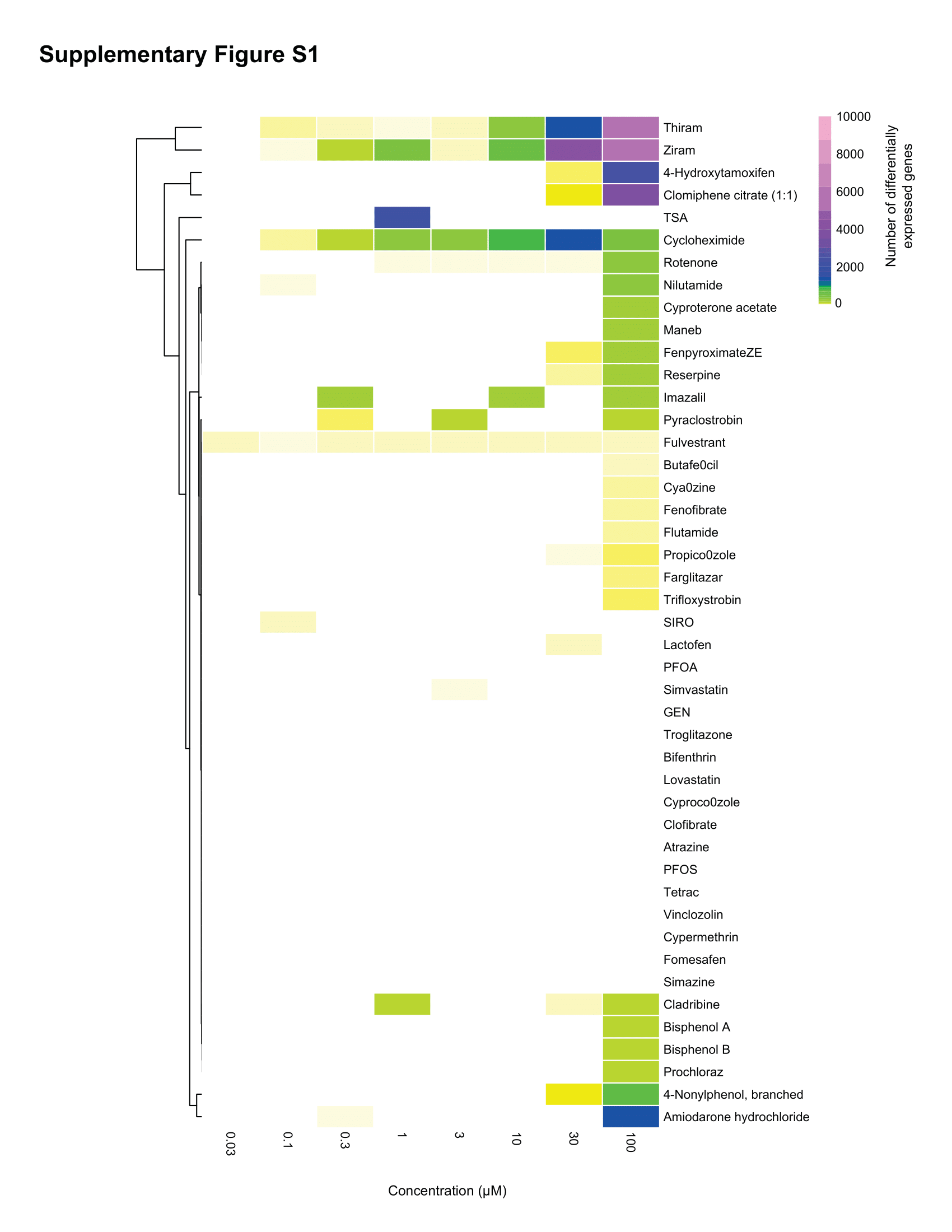

Supplement: Supplementary file 2 [file Image1.PNG]
